# Optimizing Stroke Risk Prediction: A Primary Dataset‐Driven Ensemble Classifier With Explainable Artificial Intelligence

**DOI:** 10.1002/hsr2.70799

**Published:** 2025-05-05

**Authors:** Md. Maruf Hossain, Md. Mahfuz Ahmed, Md. Rakibul Hasan Rakib, Mohammad Osama Zia, Rakib Hasan, Md. Rakibul Islam, Md. Shohidul Islam, Md Shahariar Alam, Md. Khairul Islam

**Affiliations:** ^1^ Department of Biomedical Engineering Islamic University Kushtia Bangladesh; ^2^ Bio‐Imaging Research Laboratory, BME Islamic University Kushtia Bangladesh; ^3^ Department of Computer Science and Engineering Northern University Bangladesh Dhaka Bangladesh; ^4^ Department of Neurology Kushtia Medical College Kushtia Bangladesh; ^5^ Department of Information and Communication Technology Islamic University Kushtia Bangladesh

**Keywords:** ensemble classifier, explainable artificial intelligence, feature engineering, machine learning, stroke disease

## Abstract

**Background and Aims:**

Stroke remains a leading cause of mortality and long‐term disability worldwide, presenting a significant global health challenge. Effective early prediction models are essential for reducing its impact. This study introduces a novel ensemble method for predicting stroke using two datasets: a primary dataset collected from a hospital, containing medical histories and clinical parameters, and a secondary dataset.

**Methods:**

We applied several preprocessing techniques, including outlier detection, data normalization, k‐means clustering, and missing value detection, to refine the datasets. A novel ensemble classifier was developed, combining AdaBoost, Gradient Boosting Machine (GBM), Multilayer Perceptron (MLP), and Random Forest (RF) algorithms to enhance predictive accuracy. Additionally, Explainable Artificial Intelligence (XAI) techniques such as SHAP and LIME were integrated to elucidate key features influencing stroke prediction.

**Results:**

The proposed ensemble classifier achieved an accuracy of 95% for the secondary dataset and 80.36% for the primary dataset. Comparative analysis with other machine learning models highlighted the superior performance of the ensemble approach. The integration of XAI further provided insights into the critical indicators influencing stroke classification, improving model interpretability and decision‐making.

**Conclusion:**

Our study demonstrates that the novel ensemble classifier, supported by effective preprocessing and XAI techniques, is a powerful tool for stroke prediction. The high accuracy rates achieved validate its effectiveness and potential for practical clinical application. Future work will focus on incorporating deep learning techniques and medical imaging to further improve classification accuracy and model performance.

## 1 Introduction

Stroke is a neurological disorder caused by disrupted blood supply to the brain, resulting in cell death from a lack of nutrients and oxygen [1, 2]. It is a leading global health issue, responsible for the highest rates of death and disability, affecting not just individuals but also their families and workplaces. According to the World Health Organization (WHO), there are approximately 15 million stroke cases globally each year, with around 5 million fatalities [1, 3]. Strokes can be categorized into two main types: ischemic and hemorrhagic. Ischemic strokes, accounting for about 80%–85% of cases, occur due to blocked blood flow, while hemorrhagic strokes, though less common, result from ruptured blood vessels leading to cerebral bleeding [4].

Early prediction and intervention are critical for stroke prevention. Machine Learning (ML) and data mining play essential roles in predicting strokes [5]. Risk factors for strokes can be divided into non‐modifiable factors (age, gender, race, ethnicity) and modifiable ones, including clinical conditions like hypertension and diabetes, as well as lifestyle choices such as smoking and poor nutrition [6, 7]. Individuals over 55 are more vulnerable, but strokes can occur at any age. Preventive measures include maintaining a healthy lifestyle and managing health conditions effectively [8].

The early detection of strokes is vital for effective treatment, and ML can enhance diagnosis and clinical decision‐making [9]. Numerous studies have focused on improving stroke detection speed and accuracy through ML [10]. Identifying and managing modifiable risk factors can significantly reduce the risk of stroke and related conditions like vascular dementia [11]. ML algorithms have been instrumental in computer‐assisted diagnosis (CAD) for various diseases, and advancements in artificial intelligence (AI) and ICTs are crucial for the early prediction of diseases, including diabetes and cardiovascular issues [12, 13].

### 1.1 Motivation

The need for early detection of stroke risk factors drives our commitment to developing a machine‐learning model, utilizing community data to improve patient outcomes and reduce healthcare risks.

### 1.2 Contribution

The significant contributions of our study include the following:

- We have collected extensive data from local hospitals and our neighborhoods associated with several risk factors that lead to stroke. To prepare our data before feeding to our proposed model, we have processed our data through various preprocessing techniques, including feature engineering, clustering, data normalization, cross ‐validation, etc.
- An innovative ensemble machine‐learning classifier is introduced for accurate and early‐stage stroke diagnosis. This approach achieved faster diagnoses, potentially improving patient outcomes.
- Additionally, our proposed ensemble approach demonstrates superior performance compared to conventional ML algorithms in terms of accuracy, thereby validating its effectiveness and indicating potential advancements in stroke prediction.
- Finally, we have integrated Explainable Artificial Intelligence (XAI) for key feature identification as SHAP and LIME. This approach contributed valuable insights for clinical decision‐making, enhancing outcomes.

### 1.3 Organization

The following are the remaining portions of the paper: Section Methods, 2 is an intensive literature review that offers an overview of previous studies. Section Results, 3 describes the methodology, including the research design and data gathering methodologies. Sections Conclusion, 4 and 1, 5 give the results and discussions, assessing the findings and their consequences, respectively. Finally, Section 6 concludes the study by summarizing the key findings and demonstrating future research possibilities.

## 2 Literature Review

Recent advancements in data mining and ML have significantly impacted healthcare, particularly in stroke prediction. The availability of extensive medical data has driven the development of algorithms to improve stroke prediction and treatment, offering more personalized and accurate approaches. This section reviews prior research on using ML methods for stroke prediction.

Emon et al. developed a method integrating ten classifiers with ML algorithms for early‐stage stroke prediction, achieving 97% accuracy with a weighted voting classifier and 65% with SGD. Imaging techniques like brain CT scans and MRI using (DL) may further improve accuracy [1]. Dev et al. employed a perceptron neural network on an unbalanced dataset, identifying age, cardiovascular disease, blood glucose, and hypertension as key predictors. The study lacked comprehensive validation, indicating the need for further empirical research [14].

Jamthikar et al. enhanced cardiovascular disease (CVD) risk estimation by combining ML algorithms with carotid ultrasonography, although limited by small sample size and patient focus [15]. ML algorithms have shown greater accuracy than humans in identifying stroke patients during the thrombolysis window using MRI, but larger datasets and real‐world validation are still required [16]. The introduction of “AtheroRisk‐Integrated,” a low‐cost ML model, improved cardiovascular and stroke risk assessment, increasing AUC by 18% [17]. Gaidhani et al. developed a promising CNN and DL model for stroke diagnosis, with further research needed to refine this approach [4].

The technique integrating *χ* ^2^ feature selection with Microsoft Azure ML boosted stroke prediction accuracy to 96.8%, while DL methods and larger datasets could improve precision [18]. Dritsas and Trigka achieved 98.9% AUC and 98% accuracy in long‐term stroke risk prediction but lacked short‐term insights, signaling the need for further research [3]. Despite promising accuracy rates, there is a need for improved preprocessing techniques, larger datasets, and greater use of explainable AI (XAI).

## 3 Materials and Methodology

**Figure 1:**
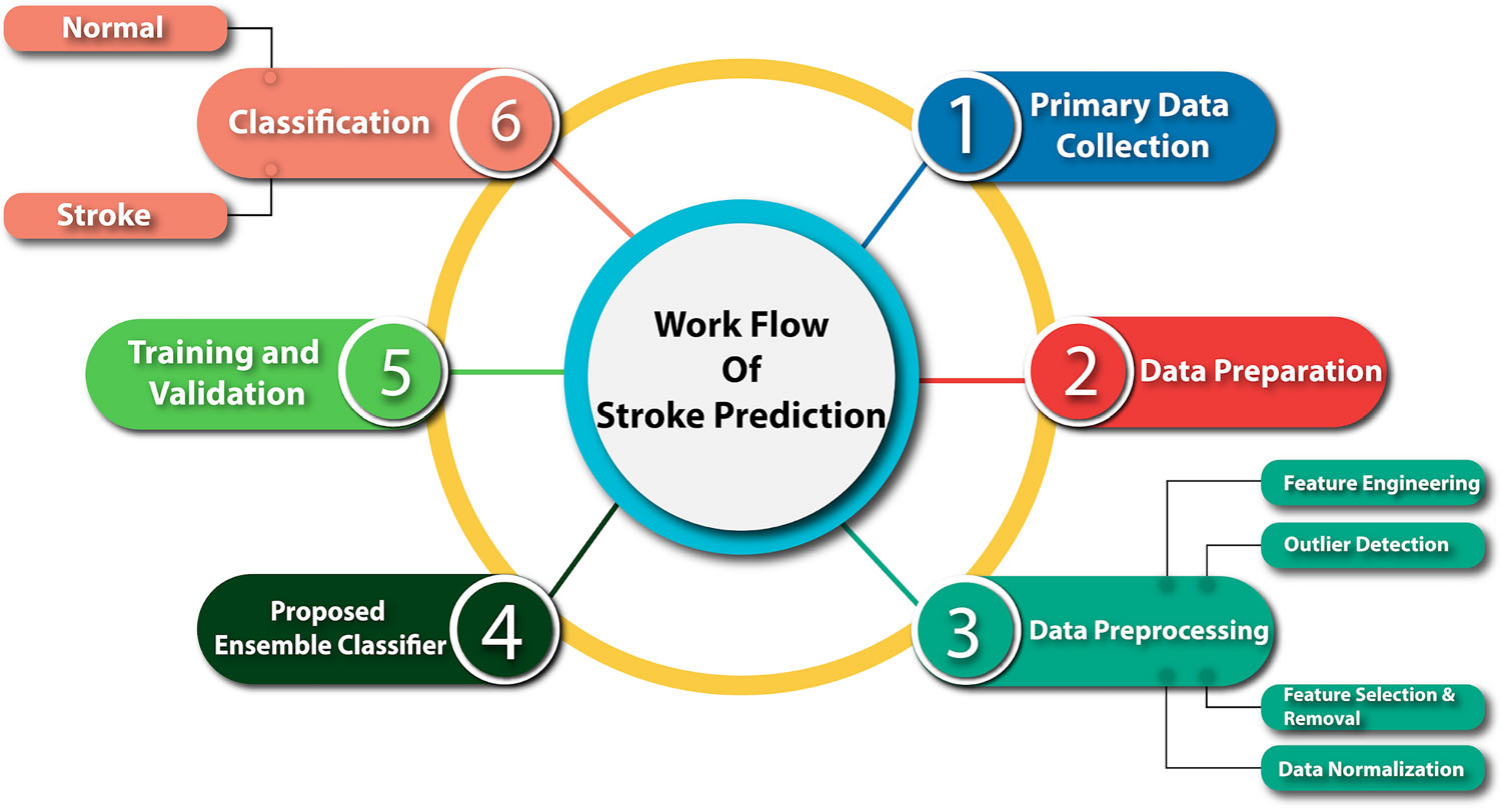
Workflow of our proposed methodology.

The system model methodology includes data collection, data preparation, data preprocessing model design, training, hyperparameter tuning, and evaluation (Figure 1).

### 3.1 Dataset

In this study, we utilized two data sets: a secondary data set, referred to as Data set 1, obtained from Kaggle, and a primary data set, referred to as Data set 2, collected from Kushtia Sadar Hospital, Bangladesh. The primary data set includes healthcare data on stroke, diabetes, and cardiovascular diseases. To ensure a comprehensive evaluation of our proposed methodology, we integrated the secondary data set to enhance the robustness of our model assessment.

#### 3.1.1 Data Set 1

Our proposed model leverages Data set 1, a secondary data set sourced from Kaggle, comprising the medical records of 5110 patients. Among them, 2115 (41.4%) are male and 2995 (58.6%) are female. Out of all samples, 4861 (95.1%) are normal patients, while 249 (4.9%) are stroke patients. The data set encompasses 11 distinct features, each corresponding to an individual patient. The features include information such as gender, age, the presence of various diseases, and smoking status. The comprehensive data set allows for a thorough analysis of the factors influencing stroke occurrence.

#### 3.1.2 Data Set 2

**Figure 2:**
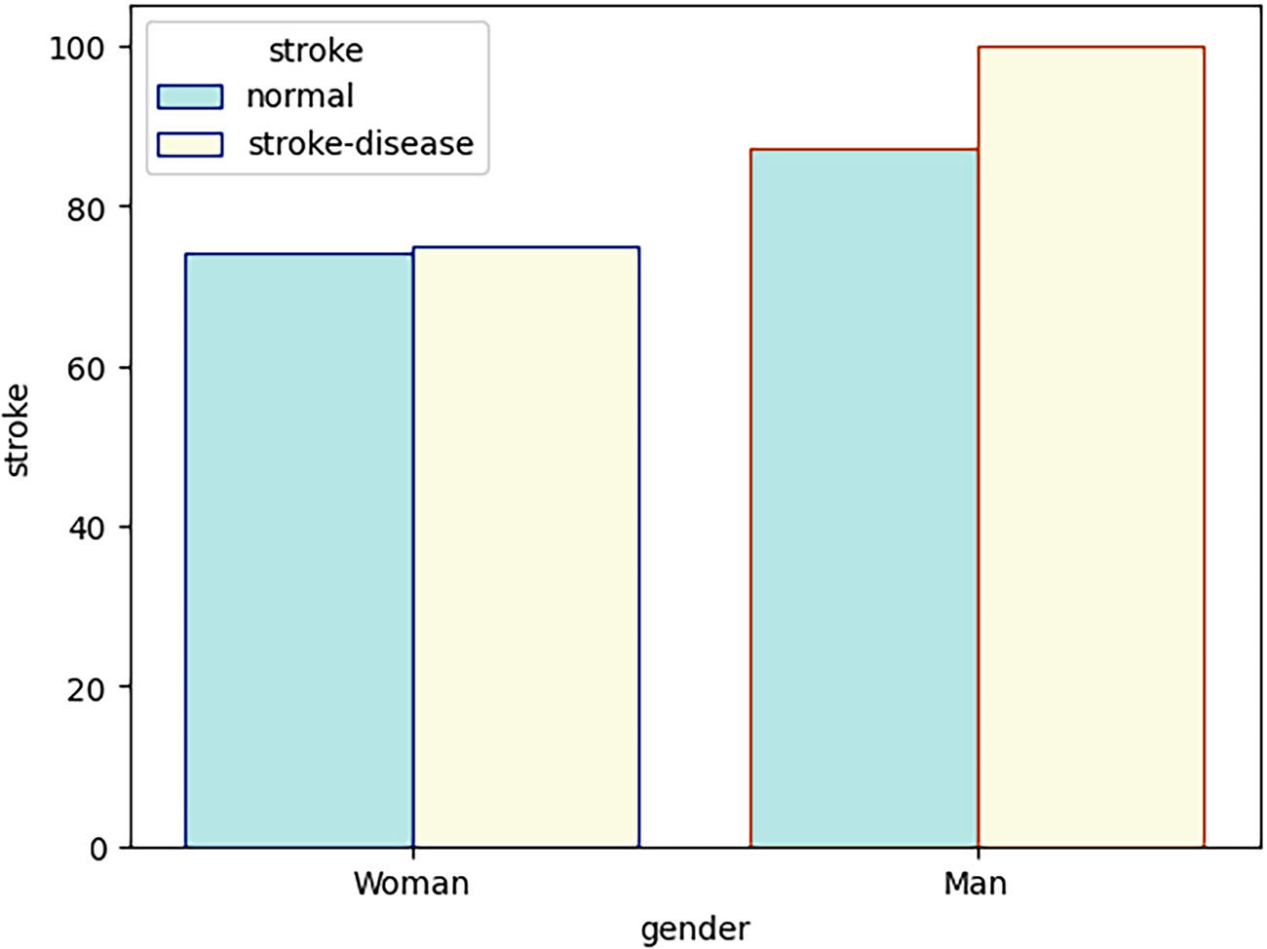
The number of diseased and non‐diseased people.

This dataset, collected from Kushtia Medical College Hospital, Bangladesh, contains 336 patient records with 20 attributes. It has a near‐even gender distribution, with approximately 55% male and 45% female, allowing for balanced analysis (Figure 2). The dataset includes 21 key features such as demographic information, medical history, clinical measurements, and diagnostic indicators. Approved by the hospital committee and reviewed by medical staff, this primary dataset, referred to as Dataset 2, provides a robust foundation for research and predictive modeling. Descriptive statistics are presented in Table 1.

**Table 1:** Descriptive statistics for Dataset 2.

Variable	Min	Max	Mean	Median	STD	Variance	MAD	RMS	Skewness	Missing
Age	0.0	1.0	0.56	0.0	0.00	0	0	0	0	0
Gender	0.0	0.0	0.0	1.0	0.49	0.247	0.493	0.746	−0.228	0
Height	5.3	1.0	62.13	63	5.38	29.032	3.547	62.364	−4.036	0
Weight	30	110	59.73	60	11.41	130.081	9.081	60.811	0.403	0
Ever_married	0.0	1.0	0.93	1	0.247	0.061	0.122	0.966	−3.529	0
Work_type	0.0	3.0	0.37	0	0.622	0.387	0.516	0.721	1.637	0
Residence_type	0.0	1.0	0.70	1	0.459	0.210	0.420	0.836	−0.873	0
Smoking_status	0.0	1.0	0.35	0.0	0.476	0.226	0.452	0.587	0.653	0
HvyAlcoholConsump	0.0	1.0	0.01	0.0	0.132	0.017591	0.035077	0.133631	7.314	0
PhysActivity	0.0	1.0	0.67	1	0.469	0.226	0.440	0.820	−0.739	0
Fruits	0.0	1.0	0.76	0.35	0.422	0.178	0.356	0.876	−1.274	0
Veggies	0.0	1.0	0.89	0.18	0.305	0.093	0.186	0.946	−2.603	0
Diabetes	0.0	1.0	0.44	0.49	0.498	0.247	0.494	0.668	0.216	0
Avg_glucose_level	4.0	31.28	8.751	7.00	4.600	21.116	3.289	9.884	2.159	0
HighBP	0.0	1.0	0.64	0.45	0.479	0.230	0.59	0.801	−0.598	0
HighChol	0.0	1.0	0.69	0.49	0.499	0.249	0.498	0.727	−0.119	0
CholCheck	0.0	1.0	0.58	0.48	0.494	0.244	0.487	0.761	−0.327	0
Heart_disease	0.0	1.0	0.68	0.42	0.464	0.215	0.429	0.761	−0.812	0
Stroke	0.0	1.0	0.52	0.49	0.500	0.250	0.499	0.721	−0.083	0
Long_time_setting	0.0	1.0	0.69	0.42	0.46	0.212	0.422	0.834	−0.858	0

### 3.2 Feature Engineering

Feature engineering involves selecting, modifying, and creating features from unprocessed data to train ML models [19]. It boosts model effectiveness by extracting relevant information and patterns [20]. ML models use feature vectors in a supervised learning approach, incorporating creation, transformation, extraction, selection, and evaluation tailored to data types and applications [21].

We employed advanced techniques to include critical factors like “BMI” and “obesity”, improving diagnostic capabilities and stroke prediction precision.

### 3.3 Outlier Detection and Removal

**Figure 3:**
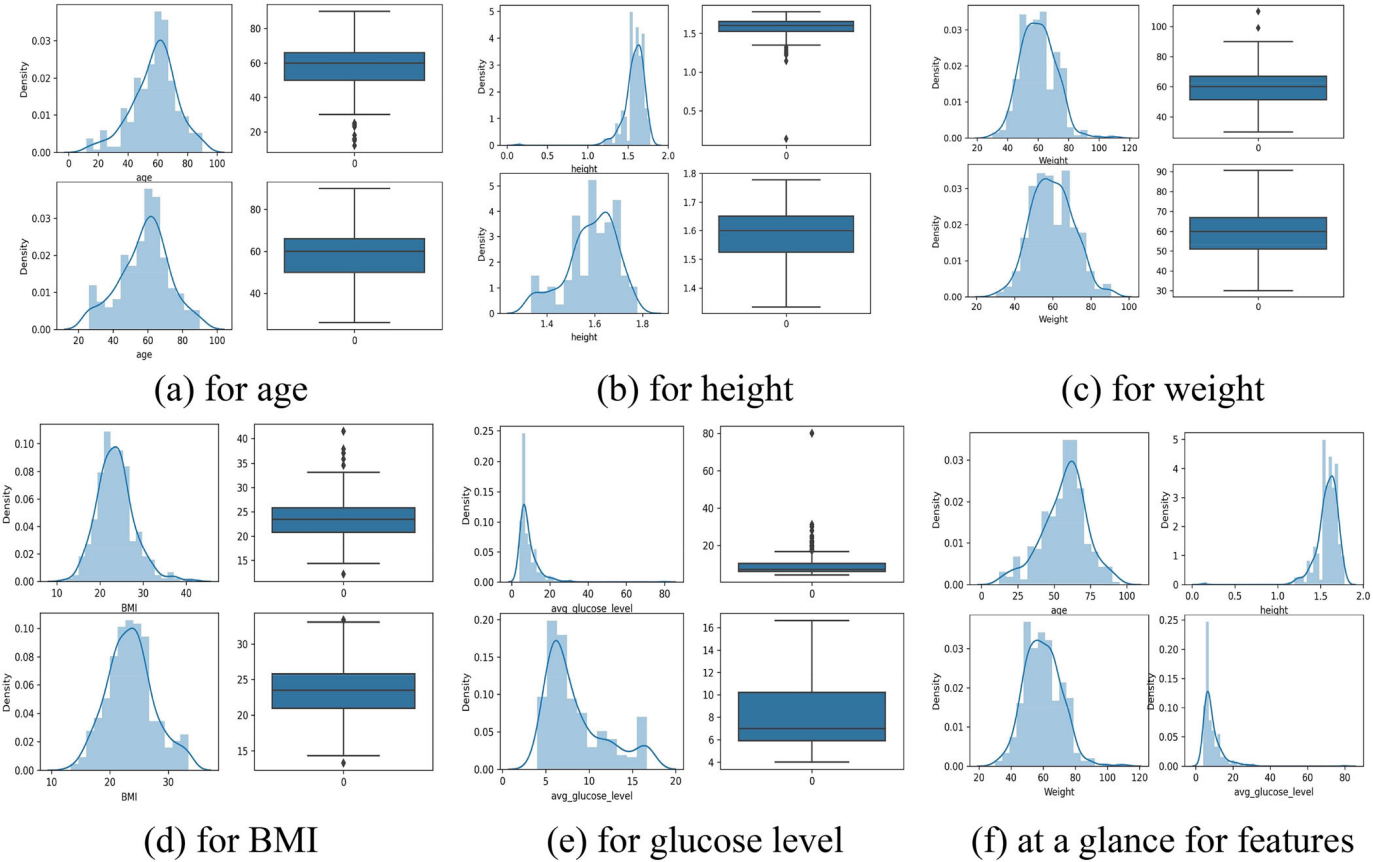
Visualization of feature distribution before and after outlier detection. (a) for age, (b) for height, (c) for weight, (d) for BMI, (e) for glucose level, and (f) at a glance for features.

Outlier detection is essential for identifying and removing data points that deviate significantly from typical patterns in a dataset. This process notably improved the accuracy of the ML system used for stroke identification [22]. We employed the Interquartile Range (IQR) method to identify outliers in age, height, weight, BMI, glucose level, and obesity, which were subsequently removed. Figure 3 illustrates the distribution of these features before and after outlier detection and removal.

### 3.4 Proposed Ensemble Classifier

**Figure 4:**
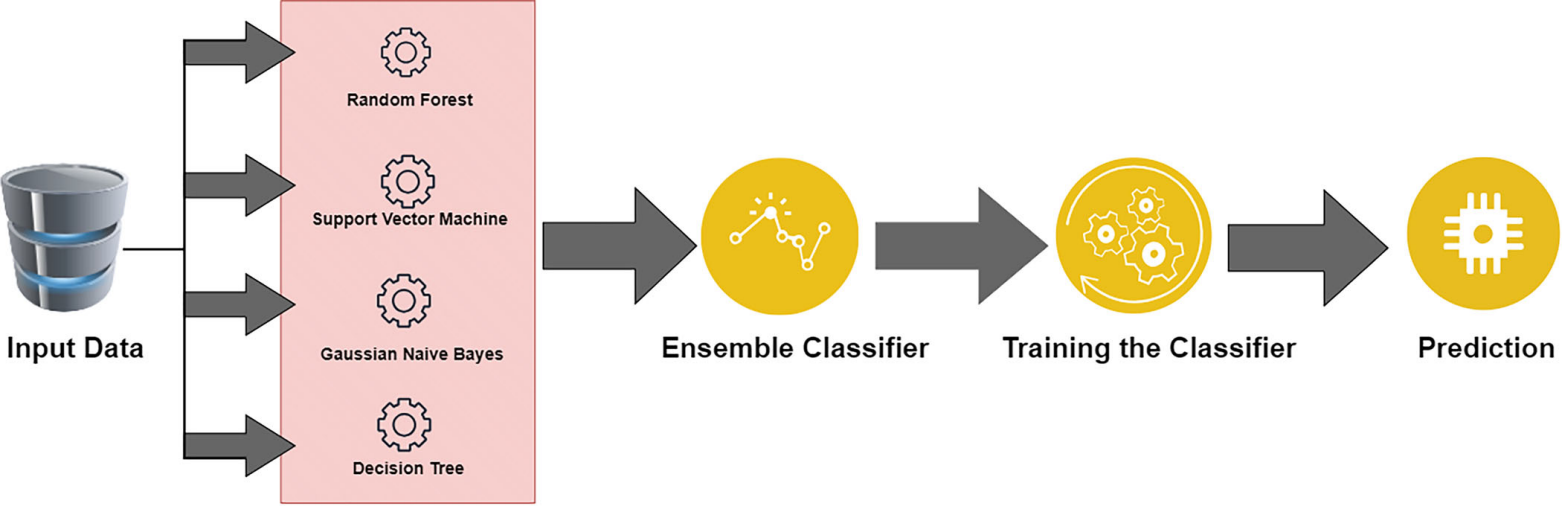
The architecture of the proposed ensemble classifier.

An ensemble classifier enhances classification accuracy by leveraging the strengths of multiple machine‐learning classifiers. Our research utilized four classifiers—AdaBoost, Gradient Boosting Machine (GBM), Multi‐Layer Perceptron (MLP), and Random Forest (RF)—to build the proposed ensemble classifier. Each classifier addresses different error patterns, and their integration improves overall performance, as illustrated in Figure 4.

AdaBoost (Adaptive Boosting) is an ensemble learning method that merges multiple “weak” classifiers into a “strong” one, minimizing training errors by weighting different datasets. Predictions are made through a weighted majority vote from the weak classifiers [23].

(1) $$F_{T}\left(x\right)=\sum_{t=1}^{T}f_{1}\;(x)$$

Gradient Boosting constructs models for classification and regression using decision trees, enhancing accuracy significantly when combined.

The Multilayer Perceptron (MLP) is a feedforward artificial neural network (ANN) with input, hidden, and output layers, using backpropagation for supervised learning [24, 25].

Random Forest classifies data by creating multiple decision trees from random training subsets and aggregates their predictions, evaluating input variable importance [26, 27].

### 3.5 SHAP as XAI

SHAP (SHapley Additive exPlanations), proposed by Lundberg and Lee [28], is a technique in XAI that provides insights into how ML models make predictions. Formally, the SHAP algorithm estimates each prediction *f(x)* as a linear function *g(x′)* of binary variables *z*′ ∈ {0, 1}*M*.

(2) $$g\left(z!\right)=\emptyset_{0}+\sum_{i=1}^{M}\emptyset_{i}z_{i}^{\prime }$$

where *M* is the number of explanatory variables.

Lundberg et al. [29] have demonstrated that the exclusive approach meeting the criteria of local accuracy, missingness, and consistency entails assigning an effect *ϕi* (known as the Shapley value) to each variable $z_{i}^{\prime }$ defined by:

(3) $$\emptyset_{i}\left(f,x\right)=\sum_{z^{\prime }\subseteq x^{\prime }}\frac{\left(\left|z^{\prime }\right|!-\left|z^{\prime }\right|-1\right)}{M}\left[f_{x}\left(z\prime \right)-f_{x}\left(\frac{z\prime }{i}\right)\right]$$

where *ϕi* is the Shapley value for feature *i*, *f* is the blackbox model, *x* is available variables, *z*′ represents the subset, *x*′ represents the selected variables, $f_{x}\left(z\prime \right)$ represents features with *i*, $f_{x}\left(\frac{z\prime }{i}\right)$ represents the without feature *i*, and $f_{x}\left(z\prime \right)-f_{x}\left(\frac{z\prime }{i}\right)$ is the deviation of Shapely values from their mean (for every single prediction). SHAP interprets feature attribution by modeling it [28]. Figure 10a,b illustrate the key features of our proposed ensemble classifier.

### 3.6 LIME as XAI

Locally Interpretable Model Agnostic Explanations (LIME) is a model‐agnostic explanation method that provides interpretable local models to clarify individual predictions from complex ML models [30, 31]. The explanations offered by LIME for each observation, denoted as x, are derived as follows:

(4) $$\emptyset \left(x\right)={\mathrm{argmin}}_{g\in G}L\left(f,g,\Pi_{X}\left(z\right)\right)+\Omega \left(g\right)$$

where *G* is the class of potentially interpretable models, *g* ∈ *G* represents an explanation considered as a model, *πx*(*z*) is the proximity measure of an instance z from x, and *Ω*(*g*) is the measure of complexity of the explanation *g* ∈ *G*.

**Figure 5:**
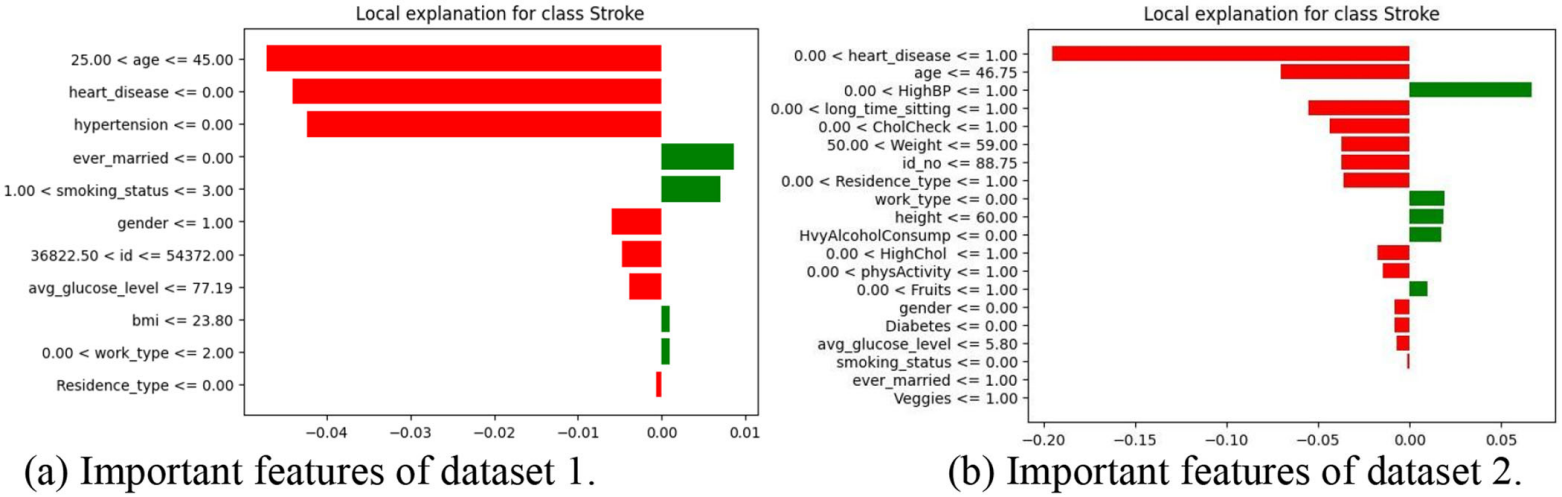
Key features identified by LIME using the proposed ensemble classifier. (a) Important features of dataset 1. (b) Important features of dataset 2.

The objective is to reduce the locality‐aware loss, denoted as *L*, without relying on any assumptions about the function *f*. It's important to note that LIME is model‐agnostic. *L* represents the degree to which the approximation *g* fails to faithfully represent *f* within the region defined by *π*(*x*). In our study, the LIME value is visualized in Figure 5 for both datasets.

## 4 Results

### 4.1 Exploratory Data Analysis

**Figure 6:**
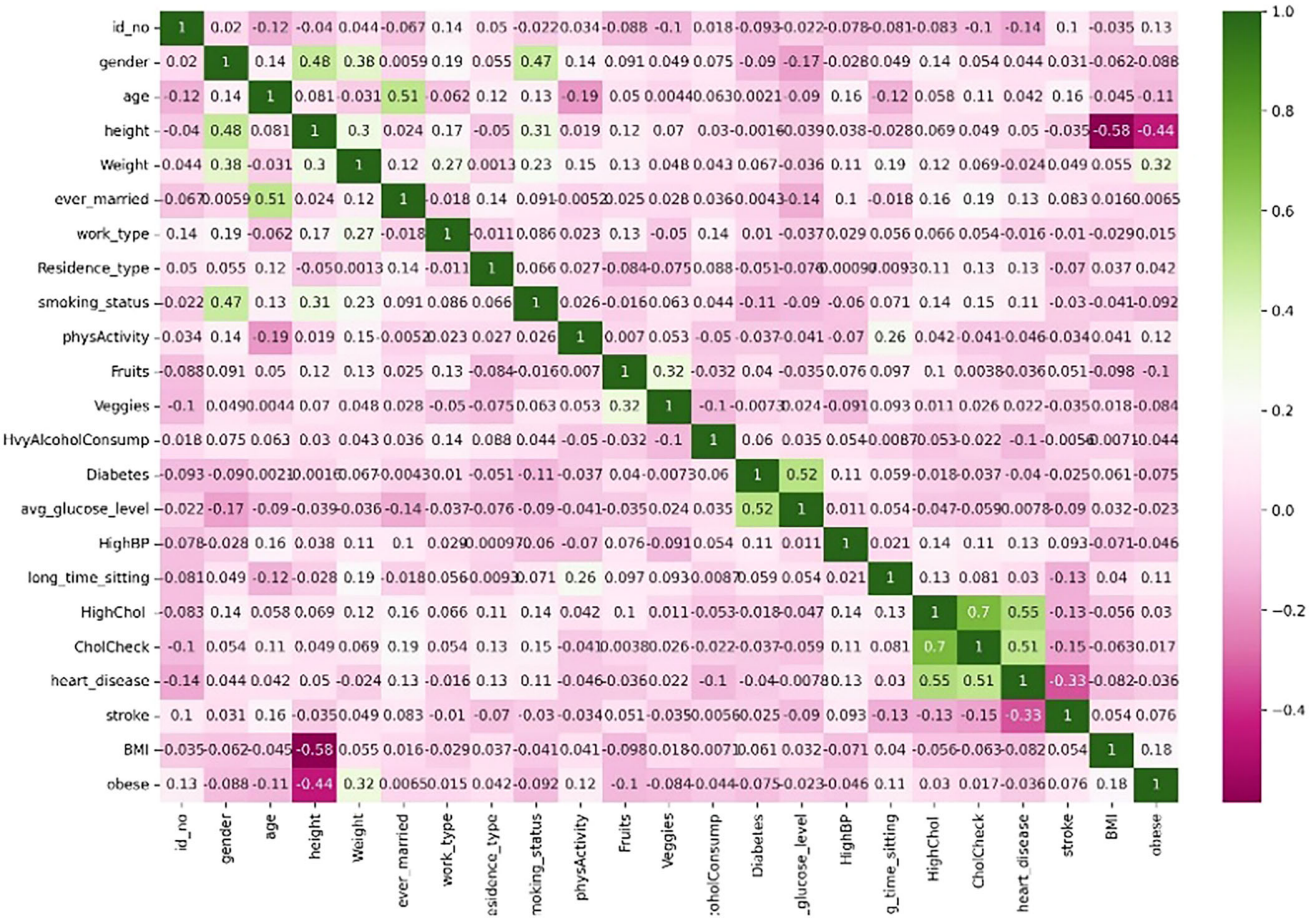
Heatmaps showing correlations among all the features of the dataset.

**Figure 7:**
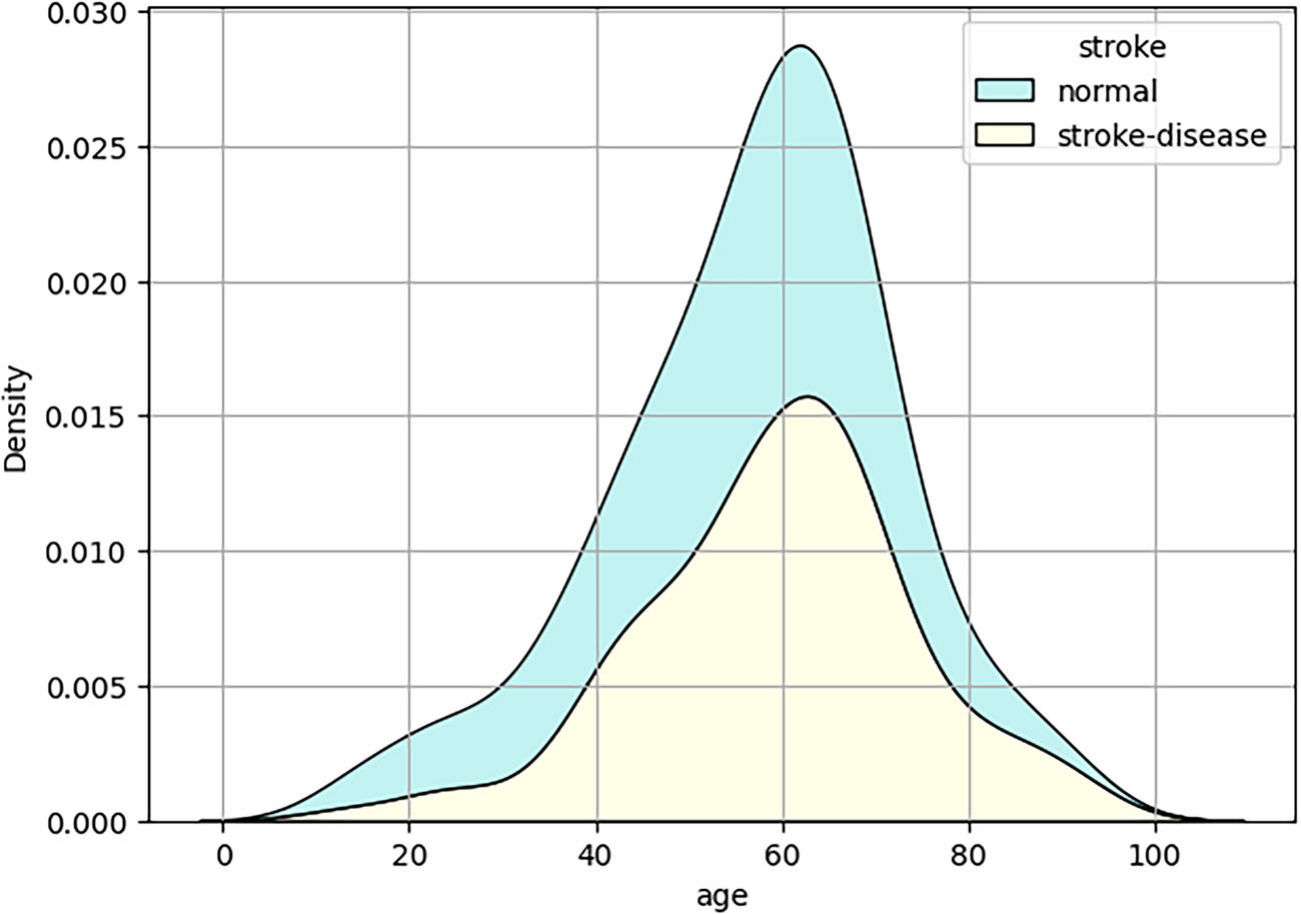
KDE plot representing diseased and non‐diseased patients based on the age distribution.

Exploratory data analysis was performed to better understand the dataset attributes, with results detailed in the following section. Figure 6 presents a heatmap showing correlations between features, where each colored cell indicates the strength of the correlation—darker colors represent stronger correlations, while neutral colors indicate no correlation. Additionally, Figure 7 displays the density distribution of patients with and without a specific illness, revealing that the affected group mainly consists of individuals aged 50–60. This highlights age as a critical risk factor for the disease, suggesting a higher likelihood of development with advancing age.

### 4.2 Experimental Results

In our experiment, we used two distinct datasets, Dataset 1 and Dataset 2, both containing a target column labeled “stroke.” We applied various classification methods, including GNB, SVM, DT, LR, CatBoost, AdaBoost, GBM, RF, MLP, XGBoost, and our proposed Ensemble Classifier. Features were processed using feature engineering methods along with outlier detection and removal.

Table 2 presents kappa statistics and Matthew's Correlation Coefficient (MCC) values for the classification systems on both datasets. Our proposed model achieved kappa scores of 92% for Dataset 1 and 61.01% for Dataset 2, with corresponding MCC scores of 89% and 61.99%. These results indicate our model's superior effectiveness.

**Table 2:** Evaluation by kappa and MCC.

Classifier algorithm	Kappa		MCC	
	Dataset 1	Dataset 2	Dataset 1	Dataset 2
GNB	0.46	0.3214	0.47	0.3233
SVM	0.56	0.3928	0.57	0.3951
Decision tree	0.73	0.3108	0.75	0.3111
Logistic regression	0.56	0.3214	0.56	0.3233
CatBoost	0.85	0.4980	0.85	0.4994
AdaBoost	0.48	0.5019	0.48	0.5071
GBM	0.65	0.5357	0.65	0.5388
Proposed ensemble classifier	0.92	0.6101	0.89	0.6199

We compared our ensemble classifier against several ML algorithms, assessing sensitivity, specificity, and accuracy. Table 3 details performance metrics based on a 70/30% training and testing split. For Dataset 1, CatBoost, RF, and our ensemble classifier attained accuracies of approximately 92%, 95%, and 96%, with sensitivities of 94%, 99%, and 99%. For Dataset 2, our ensemble classifier achieved 80.36% accuracy and sensitivity. These high accuracy and sensitivity values indicate the model's strong ability to correctly identify individuals at risk of stroke, supporting timely and reliable clinical decision‐making and potentially reducing adverse outcomes through early intervention. Table 4 reveals that LR, SVM, and AdaBoost performed lower in precision for Dataset 1, while RF, XGBoost, and our ensemble model excelled in precision for Dataset 2, with RF, CatBoost, and our model achieving high recall and *f*‐measure scores.

**Table 3:** Classification results of the different classification algorithms regarding sensitivity, specificity, and accuracy.

Algorithm	Sensitivity		Specificity		Accuracy	
	Dataset 1	Dataset 2	Dataset 1	Dataset 2	Dataset 1	Dataset 2
GNB	0.71	0.6071	0.76	0.7143	0.73	0.6607
SVM	0.82	0.6429	0.75	0.7500	0.78	0.6964
DT	0.81	0.6250	0.96	0.6875	0.86	0.6607
LR	0.9706	0.6071	0.75	0.7143	0.78	0.6607
CatBoost	0.90	0.7037	0.94	0.7931	0.92	0.7500
AdaBoost	0.75	0.6897	0.73	0.8148	0.742	0.7500
GBM	0.83	0.7143	0.82	0.8214	0.83	0.7679
RF	0.9510	0.7500	0.99	0.8462	0.95	0.7963
MLP	0.86	0.7241	0.93	0.8519	0.89	0.7857
XGBoost	0.85	0.7083	0.97	0.7500	0.90	0.7321
Proposed ensemble classifier	0.94	0.7333	0.99	0.8846	**0.96**	**0.8036**

*Note:* Bold values indicate the highest performance achieved by our proposed model compared to other machine learning models.

**Table 4:** Comparison based on precision, recall, and *F*‐measures.

Algorithm	Precision		Recall		*F*‐measure	
	Dataset 1	Dataset 2	Dataset 1	Dataset 2	Dataset 1	Dataset 2
GNB	0.79	0.6800	0.75	0.6071	0.71	0.6415
SVM	0.78	0.7200	0.75	0.6429	0.75	0.6792
DT	0.97	0.6000	0.81	0.7096	0.88	0.6122
LR	0.74	0.6800	0.81	0.6071	0.78	0.6415
CatBoost	0.95	0.7600	0.90	0.7037	0.93	0.7308
AdaBoost	0.78	0.8000	0.75	0.6897	0.75	0.7407
GBM	0.83	0.8000	0.83	0.7143	0.82	0.7547
Random forest	0.99	0.8400	0.94	0.7500	0.96	0.7925
MLP	0.94	0.8400	0.86	0.7241	0.90	0.7778
XGBoost	0.97	0.6800	0.85	0.7083	0.91	0.6939
Proposed ensemble classifier	0.99	0.8800	0.91	0.7333	0.95	0.8000

**Figure 8:**
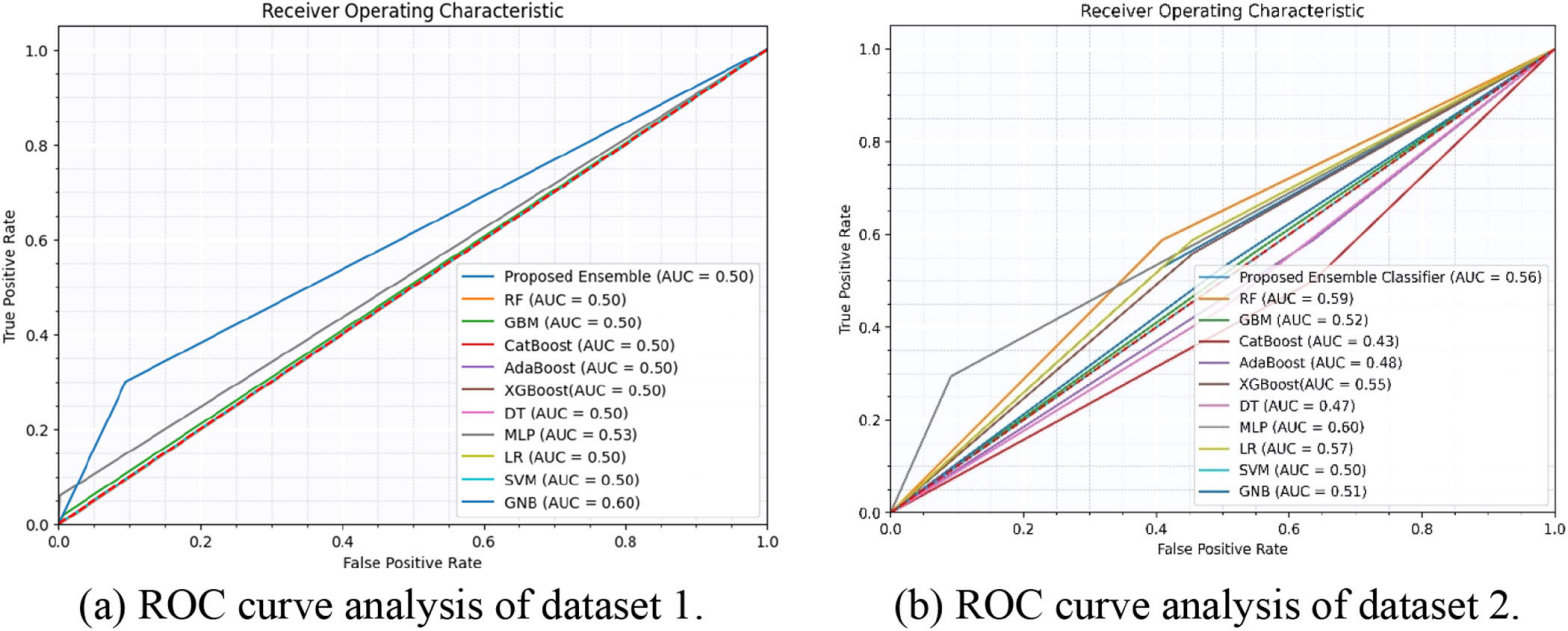
ROC curve analysis derived from the proposed ensemble classifier. (a) ROC curve analysis of dataset 1. (b) ROC curve analysis of dataset 2.

**Figure 9:**
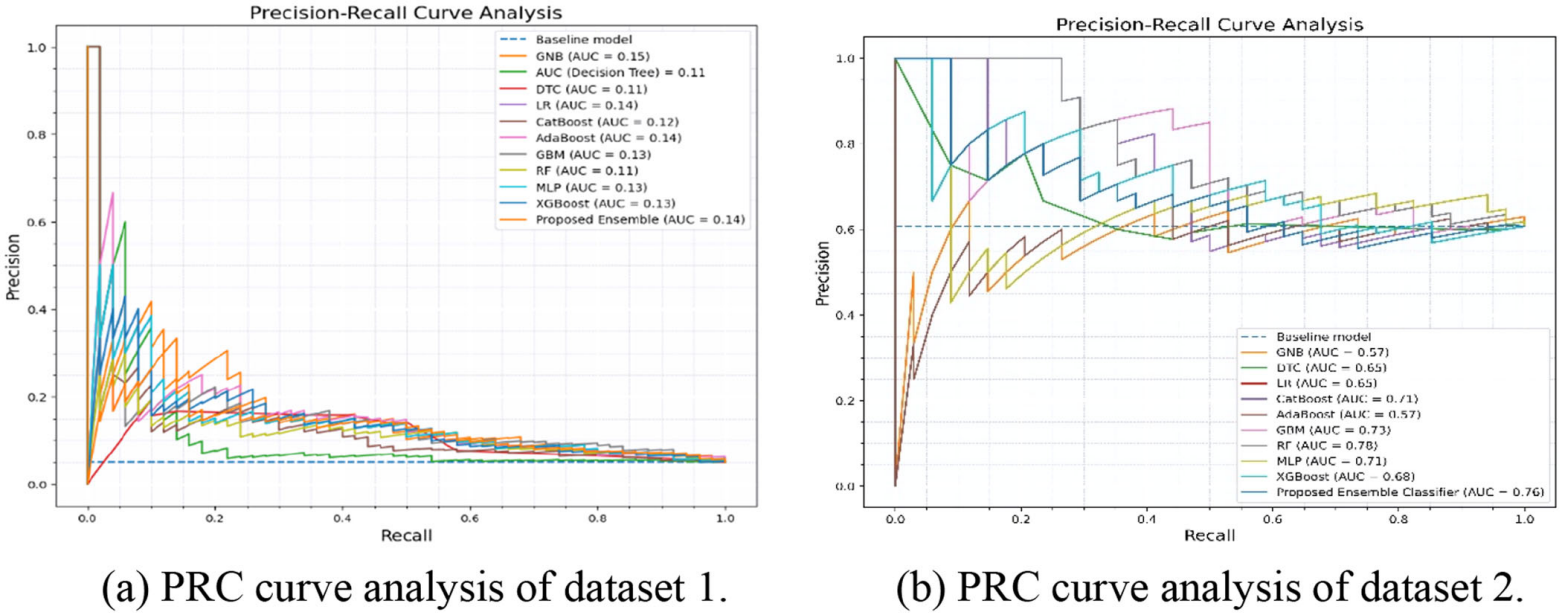
PRC curve analysis derived from the proposed ensemble classifier. (a) PRC curve analysis of dataset 1. (b) PRC curve analysis of dataset 2.

Table 5 displays the area under the ROC and Precision‐Recall Curve (PRC) values for each method, highlighting the trade‐offs between true positive and false positive rates for ROC, and precision and recall for PRC. In the Figure 8 ROC curves depict for the algorithms in Figure 8a,b for dataset 1 and 2 respectively, while Figure 9 illustrates PRC curves, visually representing the AUPRC values for both datasets. These metrics help ensure accurate stroke risk prediction by balancing detection rates and minimizing false alarms.

**Table 5:** Value of area under ROC and PRC.

Classifier algorithm	AUROC		AUPRC	
	Dataset 1	Dataset 2	Dataset 1	Dataset 2
GNB	**0.60**	0.5227	0.14	0.5794
DT	0.50	**0.5775**	0.10	0.7177
LR	0.50	0.5294	0.13	0.6213
SVM	0.54	0.5	0.10	0.5
XGBoost	0.50	0.5374	0.13	0.6265
CatBoost	0.50	0.4398	0.12	0.6361
AdaBoost	0.50	0.4612	0.14	0.5738
GBM	0.50	0.4906	0.13	**0.7544**
MLP	0.53	0.4959	0.14	0.5909
RF	0.499	0.00	0.11	0.000
Proposed ensemble classifier	0.50	0.5307	**0.14**	0.6297

*Note:* Bold values highlight the best performance, including top‐tier results in AUROC and AUPRC.

**Figure 10:**
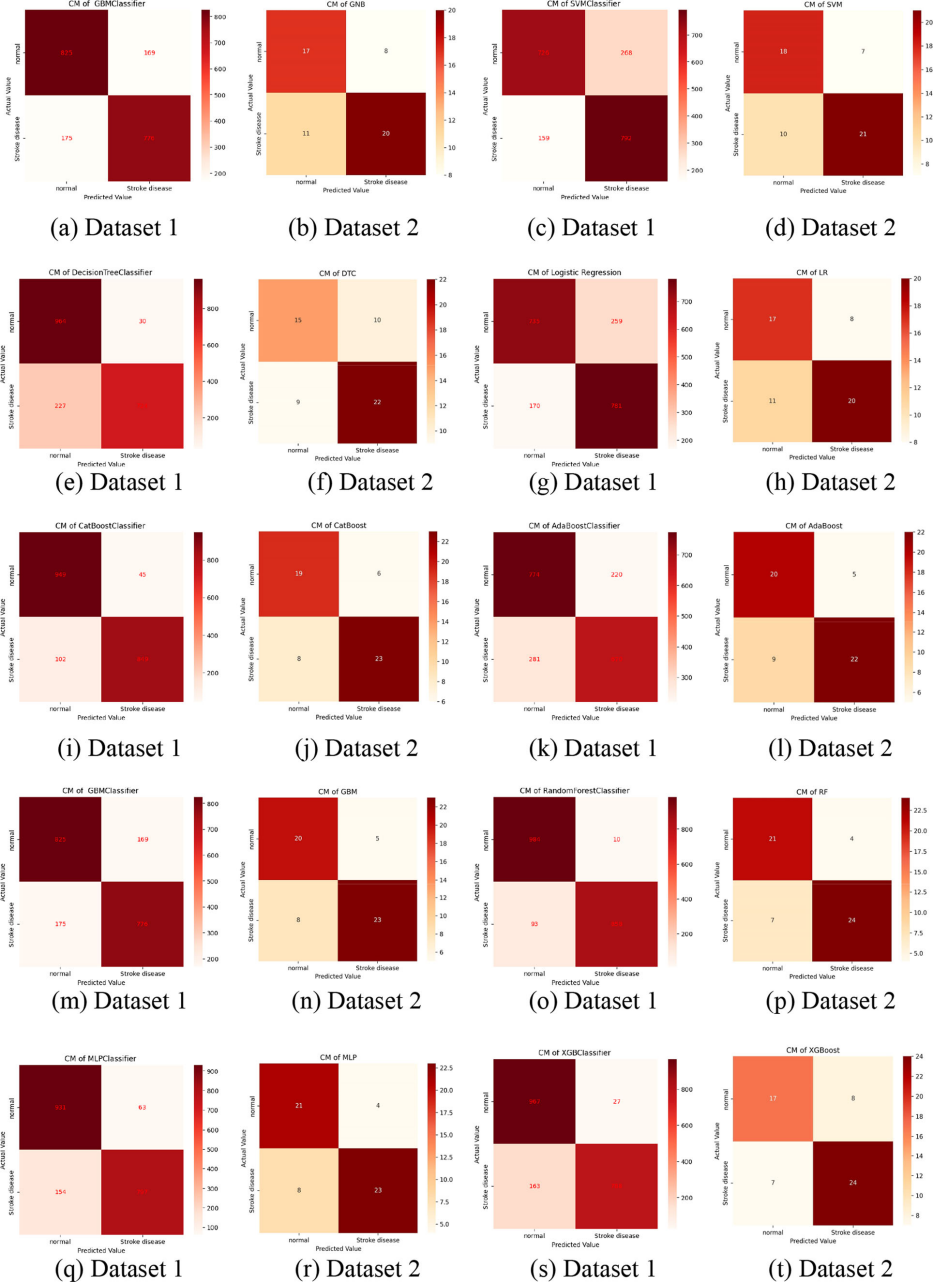
The confusion matrix of all the experimented classifiers on Dataset 1 and Dataset 2, respectively.

**Figure 11:**
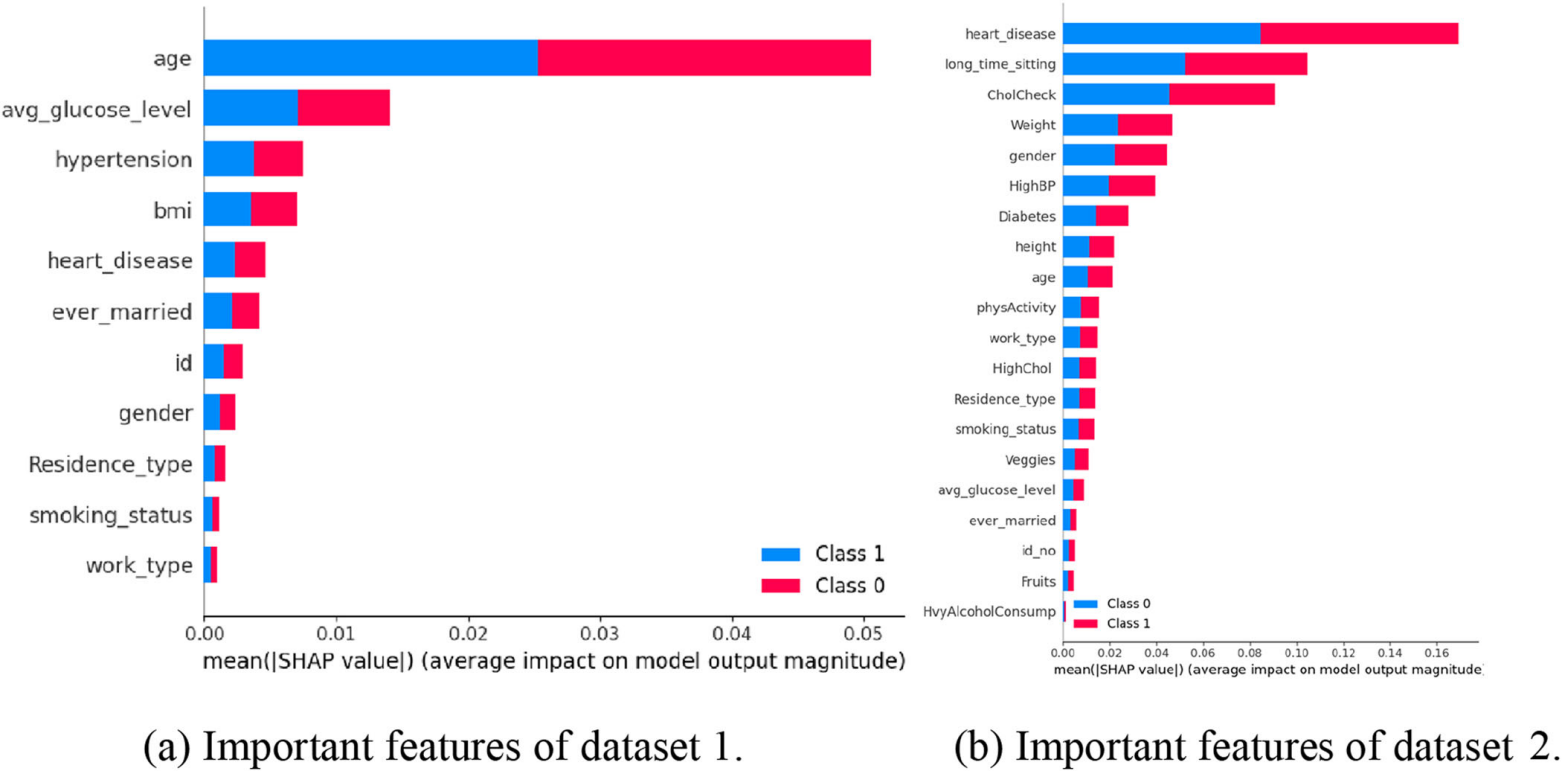
Key features identified by SHAP using the proposed ensemble classifier. (a) Important features of dataset 1. (b) Important features of dataset 2.

Figure 10 presents the confusion matrix after splitting the dataset into 70% training and 30% testing. Classifier accuracy ranged from 73% to 96% for Dataset 1% and 66.07% –80.36% for Dataset 2. We compared our ensemble classifier's performance with all ML classifiers, as detailed in Table 6. Figure 11a,b illustrate the feature ranking from our proposed ensemble classifier, highlighting key stroke risk factors.

**Table 6:** Results comparison of different ML algorithms against our proposed ensemble classifier using a fivefold cross‐validation technique.

Classifier algorithm	1st fold C (%)		2nd fold C (%)		3rd fold C (%)		4th fold C (%)		5th fold C (%)		Means (%)		STD (%)	
	Dataset 1	Dataset 2	Dataset 1	Dataset 2	Dataset 1	Dataset 2	Dataset 1	Dataset 2	Dataset 1	Dataset 2	Dataset 1	Dataset 2	Dataset 1	Dataset 2
GNB	0.952	0.4642	0.943	0.4642	0.957	0.6428	0.9437	0.6607	0.954	0.6607	0.950	0.5785	0.0050	0.0935
DT	0.994	0.4464	0.993	0.5178	0.995	0.7321	0.892	0.6428	0.959	0.5178	0.986	0.5714	0.013	0.1022
LR	0.972	0.5178	0.985	0.4821	0.973	0.7321	0.979	0.5892	0.978	0.7857	0.979	0.6214	0.0037	0.1186
XGBoost	0.950	0.4642	0.961	0.4642	0.95	0.6071	0.974	0.5892	0.965	0.7142	0.959	0.5678	0.0034	0.0947
CatBoost	1.00	0.4642	1.00	0.3928	1.00	0.5892	1.00	0.6071	1.00	0.75	1.00	0.5607	0.00	0.1235
AdaBoost	0.890	0.4642	0.910	0.4642	0.875	0.5892	0.890	0.6071	0.84	0.7142	0.881	0.5678	0.023	0.0947
GBM	1.0	0.4642	1.0	0.4642	1.0	0.5714	1.0	0.6071	1.0	0.678	1.0	0.5571	0.00	0.0832
MLP	0.200	0.5	0.00	0.5	0.20	0.6428	1.000	0.5892	1.00	0.7678	1.00	0.6	0.00	0.1001
RF	1.000	0.5178	1.00	0.4285	1.00	0.5714	1.000	0.5	1.00	0.8214	1.00	0.5678	0.000	0.1347
RF	1.000	0.5178	1.00	0.4285	1.00	0.5714	1.000	0.5	1.00	0.8214	1.00	0.5678	0.000	0.1347
Proposed ensemble classifier	0.974	0.5357	0.963	0.3928	0.968	0.5892	0.965	0.5535	0.970	0.7678	0.968	0.5678	0.0037	0.1202

## 5 Discussions

Table 4 shows our proposed ensemble classifier outperformed all algorithms in accuracy, precision, recall, and *F*1‐score. It achieved 96% accuracy for Dataset 1% and 80.36% for Dataset 2. The model's precision, recall, and *F*1‐score were 99%, 91%, and 95% for Dataset 1, and 88%, 73.33%, and 80% for Dataset 2. These strong performance metrics indicate the model's reliability in detecting stroke risk accurately, supporting effective early diagnosis and intervention. While the performance on Dataset 1 is strong, the lower accuracy on Dataset 2 highlights the challenges posed by imbalanced and limited datasets, as discussed earlier.

Feature importance can be evaluated using absolute Shapley values (in Figure 11), which are averaged across the dataset to rank features by importance. This study used an ensemble classifier to predict stroke disease, with Figure 11a showing that age is the most influential feature for Dataset 1, while Figure 11b highlights heart_disease as the key feature for Dataset 2, followed by long_time_sitting. LIME techniques reveal distinct feature importance patterns: “age”, “heart_disease”, and “hypertension” are most influential in Dataset 1, while “heart_disease”, “age”, and “HighBP” dominate in Dataset 2. These differences in feature importance may contribute to the observed performance discrepancies between the two datasets. Both SHAP and LIME provide critical insights into stroke risk prediction by identifying the most impactful factors, offering transparency in model decisions, and improving clinical decision‐making by highlighting key risk factors for early intervention.

**Figure 12:**
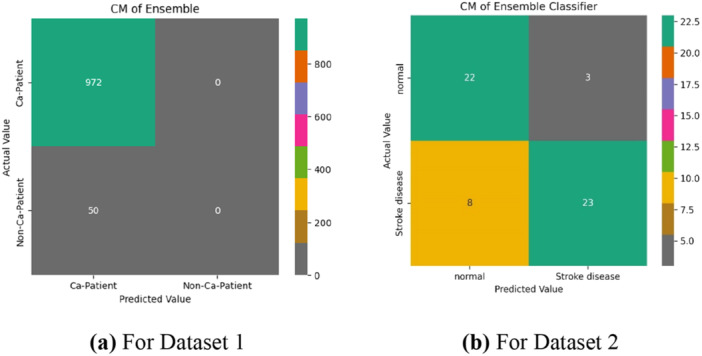
Confusion matrix of the proposed ensemble classifier trained with 80% of the data and tested on the remaining 20%. (a) For Dataset 1. (b) For Dataset 2.

The confusion matrix, illustrated in Figure 12, demonstrates the model's prediction accuracy, showing 972 correct predictions for Dataset 1 in Figure 12a and 22 for Dataset 2 in Figure 12b, emphasizing the effectiveness of preprocessing techniques in identifying stroke instances and refining the model. These matrices are essential for evaluating model performance in stroke risk prediction, helping to identify true positives and minimize false predictions, which is crucial for accurate and timely diagnosis.

**Figure 13:**
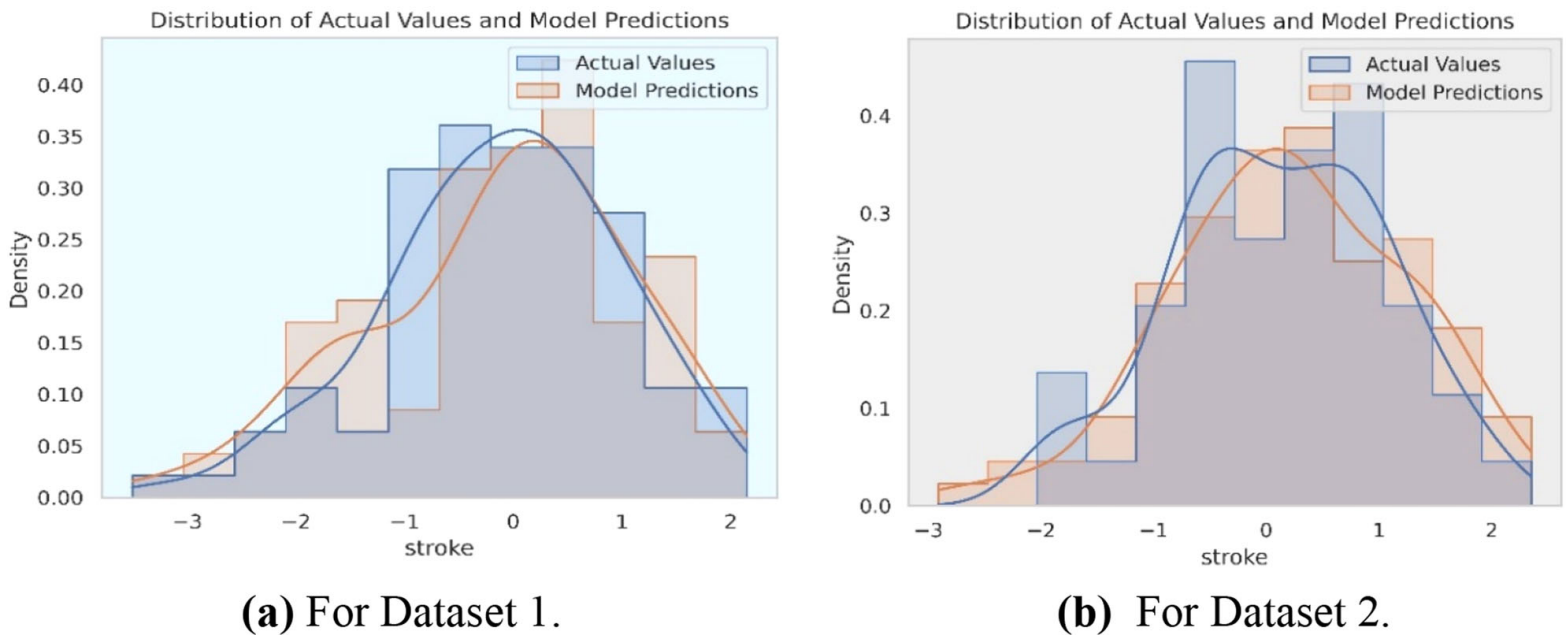
Distribution plot comparing observed values and model predictions. (a) For Dataset 1. (b) For Dataset 2.

This study has limitations: Dataset 2 showed poorer accuracy than Dataset 1, and the supervised learning approach requires extensive labeled data, which can be challenging to obtain. Figure 13 displays model performance curves. Figure 13a displays a distribution plot comparing actual values with model predictions for Dataset 1. The red curve shows actual values, while the blue curve represents the model's predictions. Their close alignment indicates the model performs well on Dataset 1. In contrast, Figure 13b for Dataset 2 reveals a noticeable discrepancy between actual and predicted values. These figures visually validate the model's predictive accuracy, helping to assess its reliability in stroke risk prediction and guiding improvements for better patient‐specific outcomes.

Our proposed ensemble model demonstrates strong performance across two datasets. On our primary dataset, it achieved an accuracy of 80.36%. To further validate its effectiveness, we tested it on a publicly available dataset, where it attained an impressive accuracy of 95%. While the accuracy on the primary dataset is relatively lower, it surpasses that of other machine learning models, and further improvements can be achieved through better optimization techniques. Overall, the results across both datasets highlight the superior performance of our proposed model.

A comparison between existing studies and our proposed approach for stroke risk prediction is presented in Table 7. Notably, the reviewed studies did not incorporate explainable AI techniques to identify and visualize the key features contributing to stroke risk. In contrast, our study integrates explainable AI methods, highlighting its distinctiveness. Furthermore, our research is based on a primary dataset, adding to the novelty and practical relevance of our work.

**Table 7:** Comparison between existing research with our proposed study.

Study	Proposed model		Precision (%)	Recall (%)	*F*1‐score (%)	Accuracy (%)	XAI used?
[32]	RF		×	×	×	78.0	×
[33]	NB		×	×	×	82.0	×
[34]	CNN‐bi‐LSTM		×	×	×	94	×
[35]	ANN		×	×	×	95.3	×
Our study	Dataset 1	Ensemble	99	91	95	96	✓
	Dataset 2	Classifier	88	73.33	80	80.36	✓

## 6 Conclusions and Future Work

In conclusion, we conducted an extensive analysis of the data collected from local hospitals and neighborhoods, focusing on various risk factors associated with stroke. Employing diverse preprocessing techniques such as feature engineering, clustering, data normalization, and cross‐validation, we prepared our data for input into a novel ensemble machine‐learning classifier. Our study presents several key findings regarding the proposed model for predicting stroke diseases. Firstly, our proposed model can successfully predict stroke diseases using the ensemble classifier technique based on its features. Secondly, our proposed ensemble classifier technique demonstrated the highest accuracy for both datasets. To assess the generalizability of our proposed model, we evaluated it on multiple datasets, including a publicly available dataset. The model achieved 80.36% accuracy on our primary dataset and 95% accuracy on the external dataset, demonstrating its robustness across different data distributions. These results indicate that our approach can adapt to varying data characteristics, increasing its potential for broader applicability. Finally, the integration of XAI through SHAP and LIME techniques, highlighting key features in stroke prediction, affirmed the model's effectiveness and furnished valuable insights for informed clinical decision‐making. This holistic approach contributes significantly to improved outcomes in stroke prediction and diagnosis. Real‐time stroke risk prediction models can be integrated into hospital systems or mobile health apps to instantly analyze patient data, alert clinicians to high‐risk individuals, and support timely medical intervention, especially in emergency or remote settings.

In the future, we will use DL approaches (CNN) to predict stroke diseases more accurately. Our focus will be increasing the classification accuracy and reducing the execution time of the model. Additionally, to mitigate the challenge of obtaining large amounts of labeled data in real‐world applications, we explored semi‐supervised and unsupervised learning approaches, which utilize both labeled and unlabeled data to enhance model performance. These methods help reduce reliance on extensive manual annotation while preserving high accuracy, making our model more adaptable and applicable to diverse scenarios.

## Author Contributions

**Md. Maruf Hossain:** conceptualization, methodology, software, validation, and writing – original draft. **Md. Mahfuz Ahmed:** data curation, writing, validation, and writing – original draft. **Md. Rakibul Hasan Rakib:** data curation, writing, and validation. **Mohammad Osama Zia:** data curation, writing, and validation. **Rakib Hasan:** data curation, writing, and validation. **Md. Rakibul Islam:** validation and writing – original draft. **Md. Shohidul Islam:** primary data providing, validation, writing – reviewing and editing. **Md. Shahariar Alam:** data curation, writing, and validation. **Md. Khairul Islam:** methodology, formal analysis, writing – reviewing, editing, and supervision.

## Ethics Statement

The dataset utilized in this study was collected from Kushtia Medical College Hospital, Kushtia, Bangladesh. We affirm that the dataset has received approval from the hospital committee and it has been thoroughly reviewed by a doctor and two staff members. The Ethical Approval Committee of Islamic University, Bangladesh, has also granted approval for the use of this dataset in research, with the reference number EC/FBS/2024/05.

## Conflicts of Interest

The authors declare no conflicts of interest.

## Transparency Statement

The lead author Md. Khairul Islam affirms that this manuscript is an honest, accurate, and transparent account of the study being reported; that no important aspects of the study have been omitted; and that any discrepancies from the study as planned (and, if relevant, registered) have been explained.

## Data Availability

The source code and the datasets used and/or analyzed during the current study are publicly available on GitHub.
